# Exploring matrix factorization techniques for significant genes identification of Alzheimer’s disease microarray gene expression data

**DOI:** 10.1186/1471-2105-12-S5-S7

**Published:** 2011-07-27

**Authors:** Wei Kong, Xiaoyang Mou, Xiaohua Hu

**Affiliations:** 1 Information Engineering College, Shanghai Maritime University, Haigang Ave., Shanghai, 201306, P. R. China; 2 Department of Radiology, Brigham and Women’s Hospital and Harvard Medical School, Boston, MA 02115, USA; 3 College of Information Science and Technology, Drexel University, Philadelphia, PA 19104, USA

## Abstract

**Abstract:**

**Background**

The wide use of high-throughput DNA microarray technology provide an increasingly detailed view of human transcriptome from hundreds to thousands of genes. Although biomedical researchers typically design microarray experiments to explore specific biological contexts, the relationships between genes are hard to identified because they are complex and noisy high-dimensional data and are often hindered by low statistical power. The main challenge now is to extract valuable biological information from the colossal amount of data to gain insight into biological processes and the mechanisms of human disease. To overcome the challenge requires mathematical and computational methods that are versatile enough to capture the underlying biological features and simple enough to be applied efficiently to large datasets.

**Methods**

Unsupervised machine learning approaches provide new and efficient analysis of gene expression profiles. In our study, two unsupervised knowledge-based matrix factorization methods, independent component analysis (ICA) and nonnegative matrix factorization (NMF) are integrated to identify significant genes and related pathways in microarray gene expression dataset of Alzheimer’s disease. The advantage of these two approaches is they can be performed as a biclustering method by which genes and conditions can be clustered simultaneously. Furthermore, they can group genes into different categories for identifying related diagnostic pathways and regulatory networks. The difference between these two method lies in ICA assume statistical independence of the expression modes, while NMF need positivity constrains to generate localized gene expression profiles.

**Results**

In our work, we performed FastICA and non-smooth NMF methods on DNA microarray gene expression data of Alzheimer’s disease respectively. The simulation results shows that both of the methods can clearly classify severe AD samples from control samples, and the biological analysis of the identified significant genes and their related pathways demonstrated that these genes play a prominent role in AD and relate the activation patterns to AD phenotypes. It is validated that the combination of these two methods is efficient.

**Conclusions**

Unsupervised matrix factorization methods provide efficient tools to analyze high-throughput microarray dataset. According to the facts that different unsupervised approaches explore correlations in the high-dimensional data space and identify relevant subspace base on different hypotheses, integrating these methods to explore the underlying biological information from microarray dataset is an efficient approach. By combining the significant genes identified by both ICA and NMF, the biological analysis shows great efficient for elucidating the molecular taxonomy of Alzheimer’s disease and enable better experimental design to further identify potential pathways and therapeutic targets of AD.

## Background

With the widespread use of the DNA microarray technology in the study of biology and medicine, large amount of gene expression data have been easily accumulated. The main challenge now is to extract valuable biological information from the colossal amount of data to gain insight into biological processes and the mechanisms of human disease. Modern signal processing and machine learning techniques provide new and efficient analysis tools to the high-throughput microarray gene expression dataset.

In the Gene expression context, similar genes exhibit similar behaviours only under a subset of conditions, not all conditions; and genes may participate in more than one function, resulting in one regulation pattern in one context and a different pattern in another. Researchers have developed various methods for clustering and identifying groups of genes or experimental conditions that exhibit similar expression patterns, such as k-means [1], self-organizing maps (SOM) [2,3] and hierarchical clustering (HC) [4]. However, these clustering algorithms suffer from two limitations, the one is they group genes (or conditions) based on global similarities in their expression profiles; the other is they only assign each gene to a single cluster. This is difficult to be interpreted by the biologists due to the large number of genes and complex underlying inter-gene dependency.

Recently, many biclustering methods have been proposed to avoid the drawbacks of the standard clustering algorithms. They can simultaneously cluster genes and conditions to obtain sets of genes that are co-regulated under subsets of conditions [5,6]. As particularly promising knowledge-based matrix factorization techniques, independent component analysis (ICA) and nonnegative matrix factorization (NMF) have been successfully applied to the biomedical applications to uncover biologically meaningful patterns from the data.

As an unsupervised analysis method, ICA uses the existence of independent facts in multivariate data and decomposes an input data set into statistically independent component. ICA-based gene classification method was first proposed by Hori,G. et al (2001) to classify yeast gene expressions during sporulation into biologically meaningful groups [7]. Liebermeister W. (2002) also presented ICA method for microarray analysis to extract expression modes, where each mode represents a linear influence of a hidden cellular variable [8]. After that, ICA has been widely used in microarray data for feature extraction, clustering and the classification on yeast cells’ cycle [9] and cancer data such as: ovarian cancer [10], breast cancer [11], endometrial cancer [12], colon and prostate cancer [13] and acute myeloid leukemia [14], etc.

For ICA decomposition, the gene expression data provided by microarray technology is considered a linear combination of basis vectors and some independent expression modes with both positive and negative expression levels. By grouping analysis on each expression mode, significant genes correspond to different single pathways can be identified.

Compare with ICA, NMF decomposes the input data as a product of two matrices that are constrained by having nonnegative elements. It is firstly proposed by Lee and Seung [15] to decompose human face images and they achieved meaningful part-based representation due to only additive, not subtractive combinations are allowed. Recently, NMF has been successfully used in feature extraction and dimensionality reduction of gene expression data. This method can simultaneously cluster genes and conditions to obtain sets of genes that are co-regulated under subsets of conditions.

However, there is no explicit guarantee to support the data has a unique representation in terms of positive factors. In the past several years, many methods have been developed to achieve further sparseness to the basis factors or encoding vectors or both of them. The sparseness of both basis factors or encoding vectors are especially welcomed in biological data analysis because both mutual independent biological process extract from gene set and efficient classification from sample set are expected. Several improvements to the standard NMF such as, local NMF (LNMF) [16], nonnegative sparse coding (NNSC) [17], sparse NMF (SNMF) [18], NMF with sparseness constraints (NMFSC) [19] and non-smooth NMF [20].

In this study, we explore the potential of both ICA and improved NMF on DNA gene expression data to identify significant genes of Alzheimer’s disease (AD). By using these methodology to decompose AD samples into expression modes (ICA) and metagenes (NMF), and combining the gene selection results from ICA and NMF, the identified genes and the underlying biological processes show many clear related pathways in AD and the activation patterns to AD phenotypes. This hence can help biologists to understand the phenotype–pathway relationship and help to find the biomarker genes for AD.

## Methods

The microarray gene expression data are traditionally represented as a *n*×*m* matrix ***X*** with *n* genes under *m* samples or conditions. The columns of the dataset denote *gene expression signatures* (GES) of *n* genes during *m* experiments or environmental conditions while each row of matrix ***X*** represent *gene expression profiles* (GEP) of each gene across all *m* conditions.

### ICA analysis on AD gene expression data

Let the *n*×*m* matrix ***X*** denote the microarray gene expression data with *n* genes under *m* samples or conditions. *x_ij_* in ***X*** is the expression level of the *i*-th gene in the *j*-th sample. Generally speaking, the number of genes *n* is much larger than that of the samples *m*, *n* >>*m*. Therefore, the transform, ***X***^T^, was often used in ICA model. Suppose that the data have been preprocessed and normalized, i.e. each sample has zero mean and standard deviation, then the ICA model for gene expression data can be expressed as:

***X***^T^**= *AS*** (1)

where, the columns of *A* = [*a*_1_, *a*_2_, …*a_n_*] are the *m*×*m* basis vectors (latent vectors) of the gene microarray data, the rows of ***S*** denotes *m gene signatures* or *expression modes*, and every row of ***S*** is statistically independent to each other. To obtain ***S*** and ***A***, the demixing model can be expressed as

***Y*** = ***WX*** (2)

**Figure 1:**
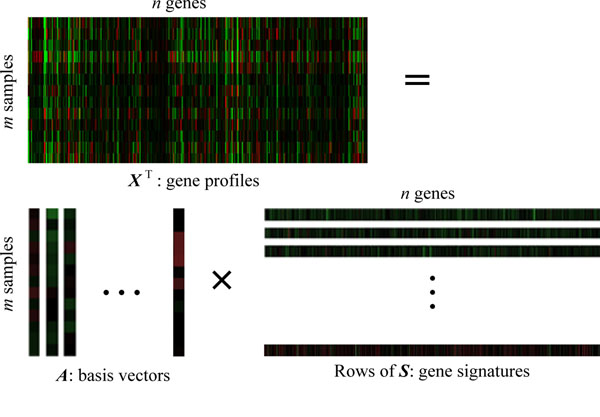
**ICA decomposes AD microarray gene expression data** X (13×6398) is AD microarray gene expression data matrix with 13 samples and 6398 genes, A is 13×13 basis vector and S is expression mode(13×6398). Here columns of A and rows of S are separated for a clear visibility.

Where ***W*** is an *m*×*m* demixing matrix. Figure 1 shows a example of ICA on AD gene expression data, detail representations will be given in the next section.

The gene expression data provided by microarray technology is considered a linear combination of independent expression modes having specific biological interpretations. The *m*-th row matrix ***A*** contained the weights with which the expression levels of the *n* genes contribute to the *m*-th observed expression profile. Hence the assignment for the observed expression profiles with different classes is valid for the rows of ***A***, and each column of ***A*** can be associated with one specific expression mode. Since the *n*-th column of ***A*** contains the weights with which *s_n_* contributes to all observations, this column should show large or small entries according to the class labels. After such characteristically latent variables have been obtained, the corresponding elementary modes can be identified to yield useful information for classification. Also, the distribution of gene expression levels generally features a small number of significantly overexpressed or underexpressed genes that form very biologically coherent groups and may be interpreted in terms of regulatory pathways.

### NMF analysis on AD gene expression data

NMF assumes the given gene expression data is combined of positive metagenes with meaningful local biological representation instead of statistically independent expression modes in ICA. This method can be applied to reduce the dimensionality of data and represent the original data as a linear combination of a reduced set of *k*-factors. The nonnegtive matrix factorization can be described as follow:

V ≈ WH (3)

Where ***V*** is a positive matrix (the microarray gene expression data) of size *n*×*m* with a desired reduced rank *k* (*k* ≤ *n*), ***W*** are *n*×*k* basis factors (also known as basis experiments [13] or metagenes [14]) with nonnegtive elements, and ***H*** contains the nonnegative coefficients (encoding vectors) of the linear combinations of the basis factors with size of *k*×*m*.

However, there is no explicit guarantee that the representation of positive factors is unique. From many improved work, non-smooth NMF (*ns*NMF) is applied in our work for the AD gene expression data since it is reported to be able to enforce sparseness to both basis factors and encoding vectors by adding a modification variable into the classical NMF model [20]. *ns*NMF is defined as:

V ≈ WSH (4)

where V, W, and H are the same as in the classical NMF model. S is a k×k positive symmetric matrix and to be defined as a “smoothing” matrix:(5)

where **I** is the identity matrix, **1** is a column vector of ones, **11**^T^ is a *k*×*k* matrix with all items are ones, and the parameter *θ* satisfies 0 ≤ *θ* ≤1. Given a positive nonzero vector ***X***, multiplying the smoothing matrix S to vector ***X***, ***SX***, if *θ* = 0, then the result is no smoothing on ***X***. If *θ* → 1, the result of ***SX*** tends to the constant vector with all elements almost equal to the average of the elements of ***X***. This is the smoothest possible vector because all entries are equal to the same nonzero value, instead of sparseness which having some large values and others close to zero.

According to the equivalently written form of Eq. (4): ***V* ≈**(***WS***)***H*** =***W***(***SH***), for a given 0 ≤ *θ* ≤1, the iteration of the algorithm can be done by substituting ***W*** by ***WS*** when updating for ***H***, and substituting ***H*** by ***SH*** when updating for ***W***. It can simultaneously sparse ***W*** and ***H*** when convergence.

**Figure 2:**
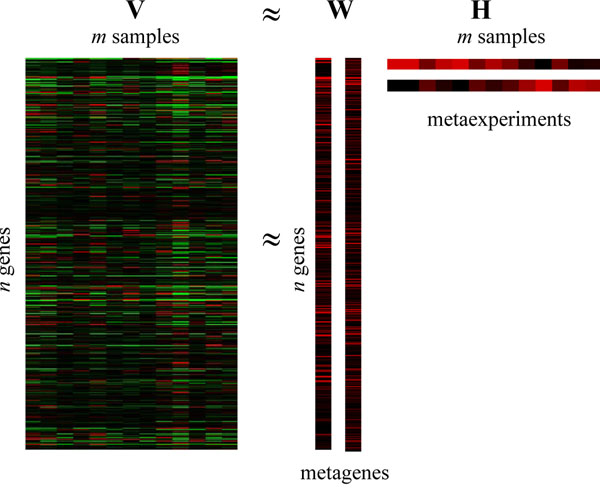
**The *ns*NMF decomposition of AD microarray gene expression data.***V* (13×6398) is AD microarray gene expression data matrix with 13 samples and 6398 genes, *W* (6398×2) is metagene matrix and *H* (2×6398) is the encoding vectors, here columns of *W* and rows of *H* are separated for a clear visibility.

Given a NMF decomposition result, we can use matrix ***H*** to classify *m* samples into to *k* classes. Each sample is clustered into a group corresponding to the most highly expressed metagenes in the sample. The nature merit of *ns*NMF is that the sparseness reinforces the genes and experiments that significantly sustain and represent the factors. Each original sample is represented by columns of basis factors with the corresponding encoding vector (the column of ***H***). It is clear that large value of both basis factors and encoding vectors play important role in representing of the original data. Figure 2 gives a NMF factorization example for the same input AD data in ICA experiment.

## Results

To evaluate ICA and *ns*NMF applied to AD DNA gene expression data, we used the data set of hippocampal gene expression of control and AD samples from GEO DataSets offered by Eric M. Blalock [21]. The hippocampal specimens they used were obtained through the Brain Bank of the Alzheimer’s Disease Research Center at the University of Kentucky. The human GeneChips (HG-U133A) of Affymetrix and Microarray Suite 5 were used in analyzing the microarray data. The procedures for total RNA isolation, labeling and microarray were described in [21] and [22]. We excluded the samples with significant noise and chose 8 control and 5 severe AD samples with 6398 genes to test FastICA(http://www.cis.hut.fi/projects/ica/fastica/) [23] and *ns*NMF method.

FastICA decomposes AD gene expression data matrix ***X*** into latent variable matrix ***A*** (13×13) and gene signature matrix ***S*** (13×6398). In FastICA algorithm, nonlinear function *g*(*u*)=*tanh*(*a*1*u) was used as the probability density distribution of the outputs during the iteration, where here *a*1 is a constant. As the FastICA algorithm relies on random initializations for its maximization and faces the problem of convergence to local optima, we iterated FastICA 100 times to alleviate the instability of the slightly different results in each iteration.

**Figure 3:**
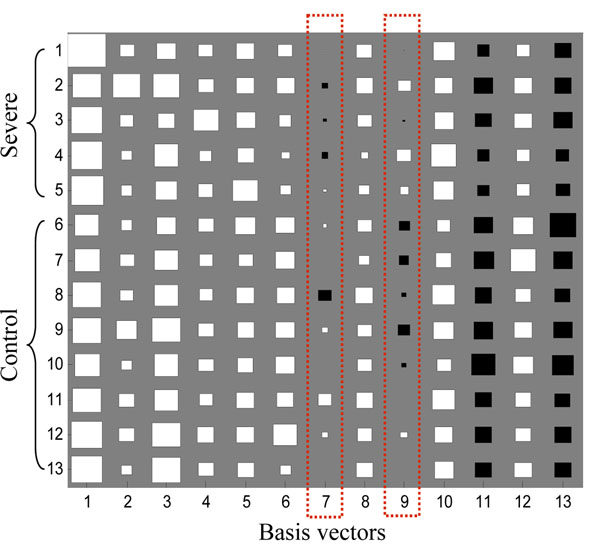
**Hinton diagram representation of latent variable matrix *A*.** The size of each square corresponds to the amount *a_nm_* of component *m* in sample *n*. White and black represent positive and negative values, respectively.

**Figure 4:**
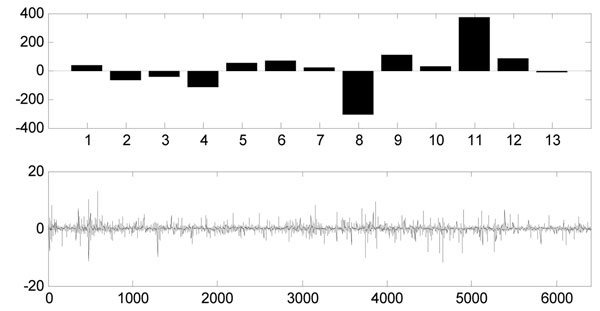
**The FastICA result of 7^th^ column of *A*.** The 7^th^ column of ***A*** which shows a strong discrimination of control and severe AD samples (upper figure) and the corresponding 7^th^ row of the expression mode (lower figure).

**Figure 5:**
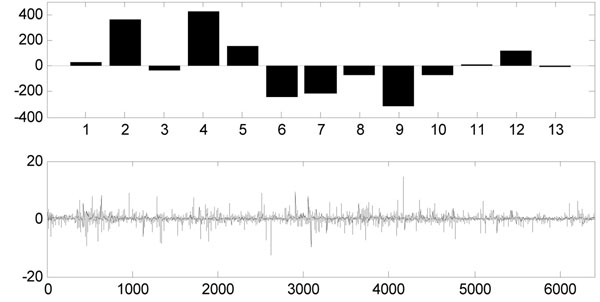
**The FastICA result of 9^th^ column of *A*.** The 9^th^ column of ***A*** which shows a strong discrimination of control and severe AD samples (upper figure) and the corresponding 9^th^ row of the expression mode (lower figure).

In FastICA result, each row of matrix ***A*** contained the weights with which the expression levels of the *n* genes contribute to the corresponding observed expression profile (row of matrix ***X***). Therefore, the profile order of rows of ***A*** is the same as that of the observed expression profiles, and each column of ***A*** is associated with the corresponding gene signature (IC). The original data set consisted of 5 severe AD microarray gene expressions (first 5 rows) and 8 control samples (last 8 rows), so this assignment is same as the rows of ***A***. Figure 3 shows the Hinton diagram representation of matrix ***A***. It is clear that the 7-th and 9-th columns of ***A*** can discriminate between severe AD and the control samples since the sign and the values are distinctly different (see Figure 4 and 5): the first 5 values (for severe AD samples) of the 7-th column of ***A*** are negative and most of the last 8 values (for control samples) are positive, and so does the 9-th column of ***A***. It can be drawn that the corresponding gene signatures (the 7-th and 9-th row of matrix ***S***) give strong contribution to the component. By giving a threshold=2.5, the positive and negative loadings correspond to up- and down-regulation of expression can be thought as significant genes for diagnosing AD. We extract more than 200 significant genes to further identification and biological analysis.

**Figure 6:**
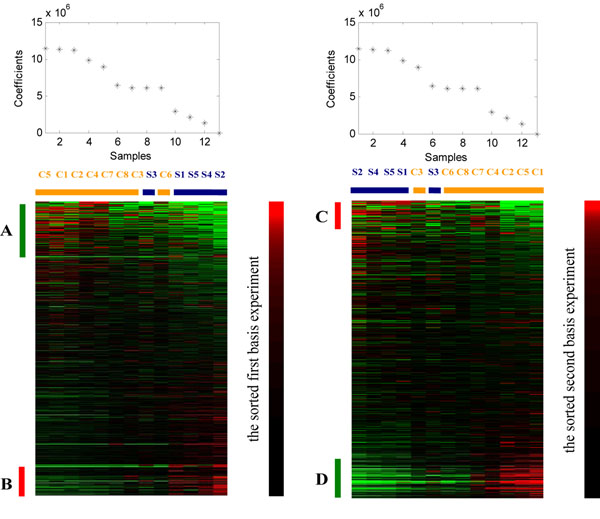
**Raw input data reconstructed by sorted *ns*NMF factors.** (a) original dataset sorted by the first metagene (the first column of ***W***) and the first metaexperiment (the first row of ***H***); (b) original dataset sorted by the second metagene (the second column of ***W***) and the second metaexperiment (the second row of ***H***). The genes marked **A**, **B**, **C**, **D** are selected gene set which significantly express in severe AD.

In *ns*NMF experiment, we ran 40 times for *k*=2, and 2000 iterations were used when convergence. Each metagene achieved by *ns*NMF is sparser and hence contains a relatively small set of genes with non-zero coefficients that determine different local gene expression features. At the same time, the encoding vectors (rows of ***H***) are used to determine the classification of the samples which are highly associated to these local biological processes. By sorting the original data set by the descending order of each metagene and the corresponding encoding vector, we can obtain the local gene expression modules. Figure 6 is the sorting results by first and second basis factor and metaexperiment respectively. The reconstructed results show that the local structures obtained are accumulated at the upper and bottom parts of the two figures which corresponding to the up-regulated and down-regulated in the severe AD. There are more than 1500 significant genes are identified by *ns*NMF which related to the pathways of AD.

## Discussion

The biologically analysis of the identified significant genes by ICA and NMF are descried as below:

From ICA factorization results, we identify more than 100 significant genes and their related pathways play a prominent role in AD and relate the activation patterns to AD phenotypes. More than 50 upregulated significant genes in severe AD were extracted in immunoreactions, metal protein, membrane protein, lipoprotein, neuropeptide, cytoskeleton protein, binding protein and ribosomal protein. Especially, in immunoreactions, genes such as: AMIGO2, BTG1, CD24, CD44, CDC42EP4, IFITM1, IFITM2, IRF7, IFI44L, IL4R, IRAK1 and NFKBIA were found upregulated in severe AD; and CD22 / MAG were downregulated. Importantly, we found the novel associations liking inflammation and the expression of APP by the molecular biological experiments. Moreover, in metal protein, ICA found many high expressed genes such as: CAMK2B, CALM1, CAPZA2, CHGB, LOC728320/LTF, MPPE1, MT1F, MT1M, MBP, SCGN, SLC24A3, SLC7A11, ZIC1, ZBTB20, ZNF500, ZNF580, ZNF652 and ZNF710. ICA also found more than 50 downregulated significant genes in above categories and some oncogenes and phosphoricproteins were low expressed in severe AD such as: CABP1, CACNG3, CAMK2B, CAMK1G, CAPZB, MET, ZNF365 and TFRC.

By studying the NMF decomposition results, one of the interesting things we found is there are not many genes they can be identified both by ICA and NMF.

By NMF, lots of metal metabolism and inflammation related genes were identified, such as, more than 230 up-regulated genes are found to be conjunction with Zinc. We also identified 321 down-regulated genes in the first metagene (the first column of ***W***) and 263 down-regulated genes in second metagene (the second column of ***W***) which are conjunction of metabolism. Compared with the control samples, there are 77 up-regulated genes, 76 down-regulated genes in first metagene and 87 up-regulated genes, 67 down-regulated genes in second metagene are conjunction of calcium (Ca (2+)). In Alzheimer's disease, calcium permeability through cellular membranes appears to underlie neuronal cell death.

Furthermore, a big cluster of genes related to inflammation are founded which are presented up-regulated in severe AD samples.

Finally, both in the factor 1 and 2 show that the genes inhibiting cell grows and the genes promoting apoptosis are up-regulated. Such as: Programmed cell death 6, deleted in liver cancer 1, cyclin N-terminal domain containing 2, killer cell immunoglobulin-like receptor, p21, p53. Whereas, those genes which speed up the cell cycle, promote cellular fission and cell repair are shown to be down-regulated.

## Conclusions

In this study microarray gene expression data have been analyzed by applying unsupervised knowledge-based matrix factorization techniques, ICA and NMF. Either the expression modes detected by ICA or the metagenes extracted by NMF, are able to assign gene into different informative biological processes which made the biclustering of genes and conditions simultaneously becomes possible.

The identified significant genes by these two methods for AD microarray gene expression data shows that few genes are the same. That means the genes with high expression level in the significant expression modes in ICA may not play an important role in metagenes in case of NMF. The reason might be they have different convergence constraints. ICA assume statistical independence of the expression modes, while NMF need positivity and sparseness constrains of metagenes and metaexperiments to generate localized gene expression profiles. According to their merits, we can expect to seek more significant genes for diagnosing an disease by applying ICA and NMF on microarray data respectively and integrating their results together.

This approach is demonstrated to be valid in our experiments. The significant up-regulation or down-regulation genes presented in the expression modes (ICA) and local molecular patterns (NMF) shows that they can classify samples efficiently and match well the clinical symptoms of Alzheimer disease. This tool can uncover meaningful biological interpretation to help biologists to understand the phenotype–pathway relationship and thus help elucidate the molecular taxonomy of AD.

## Competing interests

The authors declare no competing interests.

## Authors' contributions

Wk and XM conceived the study. WK carried out the ICA and NMF studies on the Alzheimer’s DNA microarray gene expression data and the computational analysis. XM made the biological and medical analysis of the significant genes extracted by the algorithms. XH participated in the design and help with data interpretation. All authors participated in writing and revising the final manuscript.
